# A Population-Based Approach to Mapping Vulnerability to Diabetes

**DOI:** 10.3390/ijerph15102167

**Published:** 2018-10-02

**Authors:** Stephen Linder, Dritana Marko, Ye Tian, Tami Wisniewski

**Affiliations:** 1 Institute for Health Policy, School of Public Health, The University of Texas Health Science Center at Houston, Houston, TX 77030, USA; Dritana.marko@uth.tmc.edu; 2 Formerly Novo Nordisk Inc., Plainsboro, NJ 08536, USA; slinder4496@live.com (Y.T.); wisnieta@gmail.com (T.W.)

**Keywords:** diabetes, socioeconomic factors, biological factors, cluster analysis

## Abstract

Of the 382 million people worldwide with diabetes, and if current trends continue, nearly half a billion people worldwide will have diabetes by 2035. Two-thirds of current diabetics are living in urban centers and the urban concentration of individuals with diabetes is on the rise. The problem is that in the absence of widespread clinical testing, there is no reliable way to predict which segments of the population are the most vulnerable to the onset of diabetes. Knowing who the most vulnerable are, and where they live, can guide the efficient allocation of prevention resources. Toward this end, we introduce the concept of composite vulnerability, which includes both group and individual-level attributes, and we provide a demonstration of its application to a large urban setting. The components of composite vulnerability are estimated using a novel, population-based, procedure that relies on sample survey data and nonparametric statistical techniques. First, cluster analysis identified three multivariate profiles of adult residents with type 2 diabetes, based on 35 socioeconomic indicators. Second, the undiagnosed population was screened for vulnerability based on their resemblance or fit to these multivariate profiles. Geographic neighborhoods with high concentrations of “vulnerables” could then be identified. In parallel, recursive partitioning found the best predictors of type 2 diabetes in this urban population, combined them with indicators of disadvantage, and applied them to residents in the selected neighborhoods to establish relative levels of composite vulnerability. Neighborhoods with high concentrations of residents manifesting composite vulnerability can be easily identified for targeting community-based prevention measures.

## 1. Introduction

According to the latest, official estimates, 29.1 million people have diabetes in the United States (U.S.), and 86 million have pre-diabetes [1]. The Centers for Disease Control and Prevention (CDC) notes that, if current trends continue, by the year 2050 1 in 3 Americans will have diabetes [1]. Currently, 382 million people across the globe have diabetes, and if current trends continue, by 2035, nearly half a billion people worldwide will have diabetes [2]. Two-thirds of all diabetics are living in urban centers, and the urban concentration of individuals with diabetes is on the rise in low- and middle-income countries. The human and economic burden of this disease is substantial. Worldwide, diabetes resulted in more than 5 million deaths and nearly $600 billion in health expenditures in 2013 alone [2]. These alarming increases may be the product of behavioral, economic, and demographic changes, but data on community- and region-specific characteristics are lacking [2]. Furthermore, dramatic improvements in prevention—not only in diagnosis and treatment—will be essential to reversing these trends. 

To better understand how urban environments drive rates of diabetes, the Cities Changing Diabetes (CCD) initiative launched programs across six cities (Copenhagen, Houston, Mexico City, Shanghai, Tianjin, and Johannesburg) and now extends to 17 cities. CCD was intended to support new research that would guide local coalitions to develop customized action plans. This article reports on the first phase of the Houston research, which is unique in several respects. The research design for Houston centered on the primary prevention of type 2 diabetes, targeting groups who were vulnerable to the disease but, as yet, had shown no clinical signs. The intent was to stem the tide of new cases by moving upstream to those most likely to experience onset in the future. To accomplish this, we needed new measures of vulnerability that could be applied across communities and used to identify distinctive subgroups at risk. A second phase of analysis—to be reported in a subsequent paper—would inform a new generation of interventions, better adapted to accommodating the social and cultural factors distinctive to each of these vulnerable groups. 

Two key circumstances supported this work. First, an extensive set of individual-level and neighborhood measures was available from a recent sample survey of the Houston area. These data included a number of candidate measures for defining vulnerability in biometric, social, and economic terms across the adult population. Second, recent versions of two nonparametric techniques (cluster/segmentation analysis and recursive partitioning) were now able to accommodate large data sets with more efficient algorithms. Each technique supports a distinctive but complimentary component of vulnerability, one based in profile matching and the other in predictability. Our intent is to introduce a non-parametric, population-based approach to defining and measuring vulnerability that will capture its composite features in biologic, socio-demographic and social determinants terms.

## 2. Methods

Recent research has attempted to extend the notion of vulnerability from its origins in climate change work, where the connotation was of a lack of resiliency [3] to consider screening communities for preventing or mitigating chronic disease [4]. The U.S. Centers for Disease Control and Prevention developed an index, called social vulnerability, made up of 16 variables, widely available in federal surveys [5]. These drew on four domains implicated in disadvantage: socio-economic status, household composition, minority status, and housing and transport. Moreover, the resulting data were aggregated to the census-tract level. Applications have attempted to extend these aggregate measures to characterizing subpopulations that might need special healthcare services [6]. Our approach shares the intent to screen populations using survey data and to capitalize on the consensus that social determinants are critical to health. In place of an index of multiple variables, we create a composite measurement procedure that can identify neighborhoods, drawing on individual-level data. We build from non-parametric profiles of those with the condition of interest (in our case, diabetes) to a screening process that can identify those most vulnerable to the disease by their resemblance to these profiles.

### 2.1. Data

The Health of Houston Survey (HHS) was a cross-sectional survey of randomly selected households in Houston, Texas, conducted in 2010–2011 using a complex, stratified, address-based, sampling strategy aimed to define the needs of adults and children in Houston and to identify health and socioeconomic disparities [7]. Responses from more than 5000 households were obtained via telephone, Web, and mail. The survey gathered data on general health topics, as well as clinical and socioeconomic questions pertinent to type 2 diabetes. For this study, data collected from the HHS were analyzed using cluster analysis and recursive partitioning. A public use file of the HHS data used here is available on the web and can be downloaded with a use agreement [8].

### 2.2. Nonmetric Cluster Analysis

A clustering algorithm was employed to construct profiles of the survey sample who self-reported a type 2 diabetes diagnosis (including only adults 18 years or older). Thirty-five social, economic, and behavioral factors (see Appendix A) were suggested by the general domains present in the Vulnerability Assessment coding scheme for qualitative analysis, adopted by the Cities Changing Diabetes project [9]. A 2-step procedure was employed to expand the number and type of variables included; the first step is pre-clustering to form sub-clusters that are then subjected to a more conventional, hierarchical analysis (TwoStep Cluster^©^, IBM SPSS v23, IBM, Armonk, NY, USA). This 2-step approach can simultaneously manage interval and ordinal variables and select the optimal number of clusters. Social and economic factors, often measured in categorical terms, can be “washed out” in covariance-based modeling approaches that include quantitative biological measures. These more distal factors were accommodated with a nonmetric procedure that uses the full array of variables as a profile of case-level attributes. Clusters were grouped and converged on homogeneous profile types that highlight factors that indicate vulnerability for diabetes.

A step-by-step process eliminated variables with low predictor importance from the cluster formation analysis. Whenever the predictor importance could not be used to inform further variable exclusion, the selection was guided by factor analysis conducted with all 35 variables. The process was halted when the following criteria were met: (1) The ratio of number of variables to the study sample was acceptable; (2) the cluster quality was fair or good in terms of cohesion and separation; and (3) the ratio of the largest cluster to the smallest was less than 2.

### 2.3. Screening for Matches

Each of the three clusters represents a distinct profile of person-level attributes common to a particular segment of the type 2 diabetes population in Houston. These profiles were identified as empirical types by cross-tabulating each cluster designation with the variables that were included in the final solution set [10]. The frequency distribution and location of these types were then assessed and used to screen the population without diabetes for composite vulnerability. All undiagnosed individuals were scored for cluster characteristics and weighted by the variable importance to the cluster solution. A total individual score was computed by summing scores of variables in each cluster. Percentages of people scoring in the 75th percentile or higher in each cluster were computed across 28 neighborhood areas. Scatterplots were created to select areas that had the highest percentage both across area and within cluster. 

Predictive factors identified through recursive partitioning were used to corroborate the findings from the cluster matching, based on the expectation that a higher percentage of those matching the profiles would share the predictors for type 2 diabetes, compared with those with few or no matches. Cluster profiles were applied to screen 28 candidate neighborhoods across the county for the relative density of the undiagnosed population that fit these profiles. Four neighborhoods were identified as having the highest concentration of residents matching one of three cluster profiles. 

### 2.4. Recursive Partitioning

Recursive partitioning is a statistical method for nonparametric multivariable analysis using a decision tree [11]. A total of 36 predictor variables (variables used in cluster analysis plus high blood pressure (HBP)) were applied to the classification and regression trees (CART) method, wherein an algorithm assigns data to the most homogeneous subsets according to sets of predictor variables [12].

Using a specific splitting rule called the Gini Impurity algorithm, the homogeneity of outcome variables within child nodes was measured to identify variables that produced the best binary splits starting with the root node. The recursive split process was repeated until the terminal node was reached with the highest homogeneity in the subsets. Then, a testing sample was used to find a pruned tree with the lowest misclassification rate by removing those nodes that did not improve classification. The final terminal nodes were assigned a predicted outcome weighted by the baseline event rate in the root node to account for unbalanced data. To find the optimal tree with the lowest misclassification rate, 10-fold cross-validation was performed to measure the misclassification rate of each tree. In this process, the original data set was randomly divided into 10 sub-data sets of the same size, then one sub-data set was used to validate a tree trained by the other nine sub-data sets and this was repeated 10 times. Variable importance for each predictor was calculated by adding Gini Impurity scores generated by that predictor in nodes, which acted as a splitter or surrogate. Results were rescaled and the top predictor was given a relative score of 100. The analysis was conducted with the Salford Predictive Modeler^®^ (Salford-Systems, San Diego, CA, USA). 

## 3. Results

### 3.1. Cluster Analysis

Cluster analysis identified 11 of the 35 potential vulnerability factors as important indicators of type 2 diabetes. Similar to recursive partitioning, the relative importance of these indicators is defined by importance weights: health insurance (1.0), age (0.76), participation in public programs (0.72; in the US, this indicator captures participation in state and federal safety-net programs based on income eligibility, such as food and housing assistance, medical insurance, and income subsidies), employment (0.55), Federal Poverty Level (FPL) category (0.39) (in the U.S., an economic measure used to decide if household members, based on household income and number of persons, qualify for a range of federal programs), degree of difficulty buying food (0.32), number of days with self-reported “poor” health (0.29), race/ethnicity (0.27), general health status (0.12), social support category (0.03), and physical activity level (0.03). 

**Figure 1:**
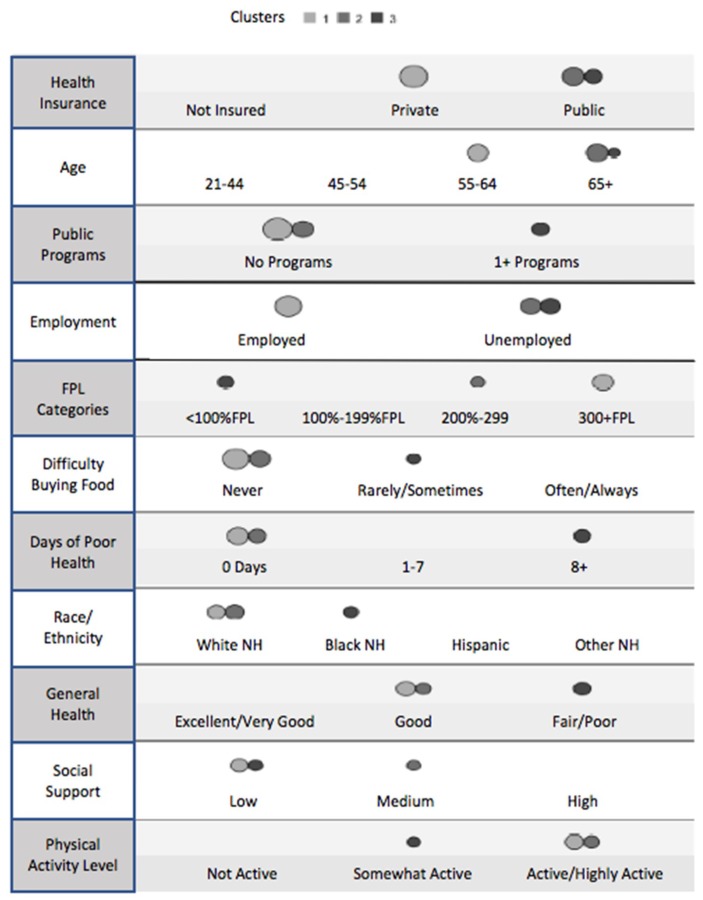
Cluster comparison describing the proportion of respondents for each indicator within a cluster. FPL—Federal Poverty Level, an economic measure used to decide if household members (based on household income and number of persons) qualify for a range of federal programs in the U.S.; NH—Non-Hispanic. Circle size corresponds to the proportion of cases that fit that indicator for each cluster.

Based on these 11 indicators, 352 of the 431 respondents with self-reported type 2 diabetes were classified into three unique clusters. The three distinct profiles are shown in Figure 1. The indicators appear in order of relative influence, and the size of the circles corresponds to the proportion of cases that fit that indicator for each cluster. As displayed in Table 1, 174 (49.4%) were in cluster 1, 100 (28.4%) were in cluster 2, and 78 (22.2%) were in cluster 3.

In cluster 1 (49.4%, *n* = 174), most of the individuals with type 2 diabetes were aged 55–64 years (50.6%), employed (78.2%), and white non-Hispanic (37.4%), although it is worth noting that the Hispanic population represented 28.2% of this cluster. The majority had private health insurance (86.8%) and was not eligible or did not participate in any public programs (98.3%). Slightly fewer than half (48.9%) lived at or above 400% of the FPL. The majority (82.8%) had never had difficulty buying food, an indicator of economic hardship, and 36.2% were in the “low social support” category. Regarding overall health, 51.7% reported 0 days of “poor” health, 43.1% described their general health status as “good,” and 40.2% were active or highly active.

In cluster 2 (28.4%, *n* = 100), 96.0% of those with type 2 diabetes were aged 65 years or older, 81.0% were unemployed, and 71.0% identified as white non-Hispanic. All respondents had public health insurance (100.0%), 94.0% were not eligible for, or did not participate in, any public programs, and 39.0% lived at 200% to 399.9% of the FPL. Most never had difficulty buying food (92.0%) and 38.0% considered the amount of social support they received as “medium”. Compared with cluster 1, more individuals in cluster 2 reported 0 days of “poor” health (61.0%), described their general health status as “good” (47.0%), and were active or highly active (49.0%).

In cluster 3 (22.2%, *n* = 78), 37.2% of those with type 2 diabetes were aged 65 years or older and 56.4% were black non-Hispanic. The majority was unemployed (98.7%), had public health insurance (80.8%), participated in one or more public programs (78.2%), and lived below 100% of the FPL (64.1%). Approximately half of individuals in cluster 3 reported rarely or sometimes having difficulty buying food (50.0%) and considered the level of social support that they received to be “low” (55.1%). Overall, cluster 3 had relatively worse health than clusters 1 and 2; 69.2% of these respondents reported eight or more days of poor health, 76.9% described their general health as fair or poor, and 44.9% were somewhat active.

### 3.2. Recursive Partitioning 

To identify factors that were predictors of diabetes, a recursive partitioning algorithm was applied to data gathered from 4749 participants in the HHS. Of all survey respondents, 9.1% (*n* = 431) reported having a diagnosis of type 2 diabetes. The initial CART model (Model 1) considered HBP, age, body mass index (BMI), and general health as explanatory variables but finally characterized respondents on the presence of HBP only. Of 1611 respondents with HBP, 320 (19.9%) also had diabetes; of 3138 respondents without HBP, 111 (3.5%) also had diabetes. This model was defined as the best-fit. Variable importance of the four predictors was ranked: HBP was the most important (100), followed by age (80.4), BMI (65.5), and general health (62.4). The misclassification rate was 29.52%, indicating that predictive accuracy of this model was 70%.

Then, three models were developed with different combinations of the top predictors from Model 1 [12]. Model 2 excluded blood pressure and determined age and BMI to be the most relevant predictors of type 2 diabetes. Of 2000 respondents who were between 20 and 44 years of age, only 41 (2.0%) had diabetes. Of 1409 respondents aged 45 years or older with a BMI > 26.93 kg/m^2^, 306 (21.7%) had diabetes. In comparison, of the 1340 respondents aged 45 years or older with a BMI ≤ 26.93 kg/m^2^, only 84 (6.3%) had diabetes. Variable importance of the predictors was ranked: age was the most important (100), followed by BMI (46.0), and general health (41.4). The model accuracy was 74.14%. 

Model 3 excluded BMI; in this model, as in Model 1, HBP was singularly predictive of diabetes. Model 4, which excluded HBP and BMI, characterized respondents based on age and general health status. As in model 2, of 2000 respondents who were between 20 and 44 years of age, only 41 (2.0%) had diabetes. Of 1589 respondents aged 45 years or older who described their health as good, fair, or poor, 322 (20.3%) had diabetes. Of 1160 respondents aged 45 years or older who described their health as excellent or very good, 68 (5.9%) had diabetes. Age (100) was followed by general health (41.4) in terms of variable importance, and model accuracy was 70.98%. In sum, three variables consistently emerged as the most important predictors of diabetes in the Houston population: HBP, age ≥ 45 years, and BMI > 26.93 kg/m^2^, with health status following closely behind. 

### 3.3. Screening the Undiagnosed for Profile Matches

**Figure 2:**
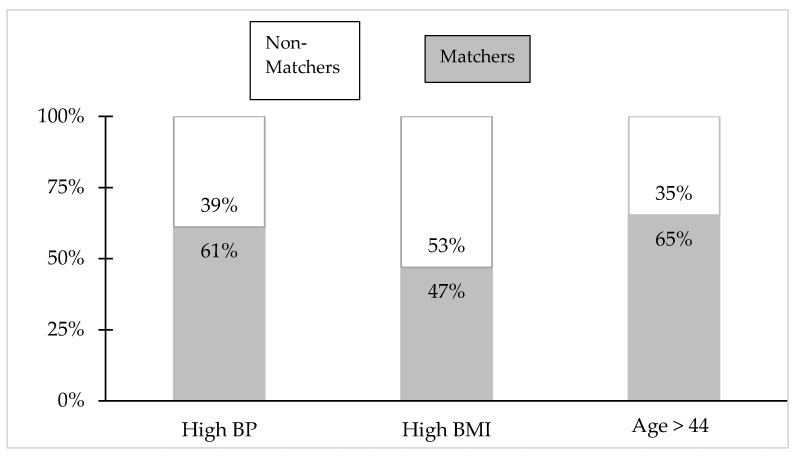
Comparison of those who match cluster profiles (Matchers) vs. those who do not (Non-Matchers), along the three best biological predictors of diabetes. Best predictors based on the recursive partition analysis. BMI—body mass index; BP—blood pressure; population weighted data.

The matching of those without diabetes to cluster profiles was accomplished by scoring individuals for each indicator match, summing the scores (weighted for importance), and designating the 75th percentile and above as the subpopulation that comes closest to one of the three profiles characterizing people with diabetes. Before this resemblance in social and economic terms can be translated into vulnerability, it must be considered whether biological predictors of diabetes corroborate the profile matching. Figure 2 compares the matching and nonmatching subpopulations relative to the best three predictors found with recursive partitioning. The matchers are more likely to have HBP (61%) and to be older than 44 years of age (65%). For high BMI, the matchers and non-matchers are close to even (47% vs. 53%). These are population-weighted percentages. If we look at the raw respondent data (unweighted), then matchers are higher on all three, consistent with the results of the recursive partitioning which can accommodate only unweighted data. 

### 3.4. Defining Vulnerable Neighborhoods

**Figure 3:**
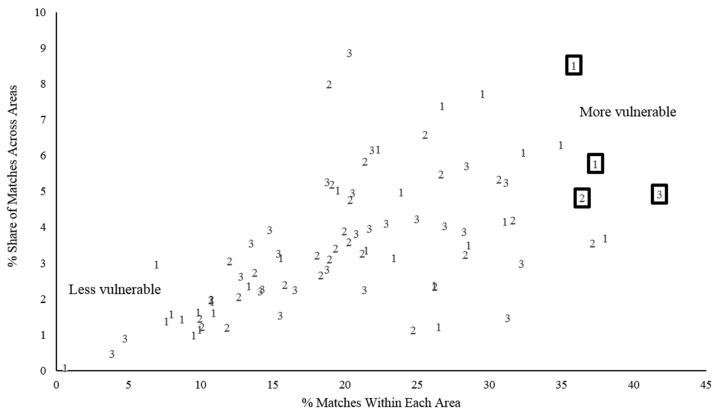
Areas by percentage match to the three cluster profiles (population weighted data).

Those who do not have diabetes, but match the profiles of people with diabetes, are not uniformly distributed across the county. Notably, high concentrations of residents who match profile characteristics can be mapped to identify neighborhoods that are especially vulnerable. Two dimensions of concentration are of interest. The first is the proportion of the total subpopulation of profile matchers who reside within a given area; this indicates the relative share. The second is the proportion of an area’s population made up of matchers; this indicates a relative degree of vulnerability—the higher the proportion of matchers, the greater is the area’s relative need for prevention. Following the Health of Houston protocol, 28 ZIP Code™ aggregation areas within Harris County, Texas, were considered. Figure 3 shows the distribution of the 28 areas along the two dimensions of interest for each of the three cluster profiles. The four areas (appearing in boxes) are among the highest in concentration of matching residents and fall within the highest quartile in area-wide share.

**Figure 4:**
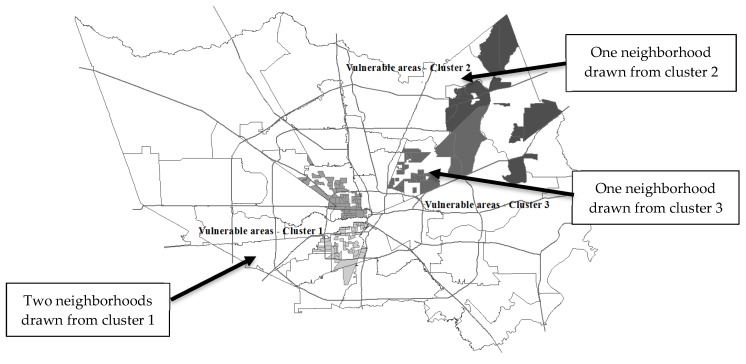
Most vulnerable areas, based on % residents who match cluster profiles.

These represent the most vulnerable neighborhoods compared with others in the county, and geographically represent the spatial concentration of people who match the cluster profiles of those diagnosed with diabetes (Figure 4). The four neighborhoods identified are widely different in sociodemographic composition and include neighborhoods of disadvantaged African Americans, middle-class Latinos, affluent white Americans, and working-class ethnically mixed neighborhoods.

### 3.5. Composite Vulnerability

Within these four neighborhoods, the vulnerability estimates can be further refined by overlaying the three best empirical predictors of diabetes, derived from recursive partitioning, for this general population: being older than 45 years of age, having HBP, and a high BMI. Those within vulnerable neighborhoods who have two or more of these characteristics take on an added level of physical vulnerability. Similarly, a third set of indicators can be incorporated for capturing a more traditional aspect of vulnerability—social and economic disadvantage. Again, three indicators were selected that each reflect different aspects of disadvantage: difficulty paying rent or buying food, living below 200% of the FPL, and dependence on public assistance. Those with two or more of these factors present can be said to experience financial vulnerability. 

**Figure 5:**
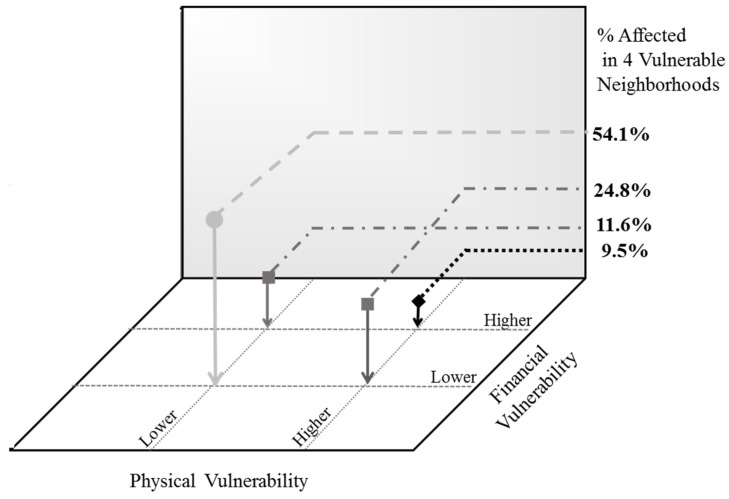
Composite vulnerability (population weighted data).

Physical vulnerability was plotted against financial vulnerability for the four most vulnerable neighborhoods (Figure 5). Those who reside in vulnerable neighborhoods, who are disadvantaged, and who have the key physical factors that predict diabetes in the population warrant the most attention to prevent the onset of the disease; these are reflected by the black diamond. The light grey circle, in contrast, represents those at lower levels of vulnerability on both of these dimensions. Together, these four points represent different levels along a spectrum of vulnerability. The relative composition of the neighborhoods is shown on the vertical axis. The majority of those within vulnerable neighborhoods (54.1%) manifest neither the physical nor the financial markers traditionally associated with the risk of type 2 diabetes, but about 40% of the residents in these neighborhoods will match the profile of those already diagnosed with diabetes.

## 4. Discussion

Our vulnerability estimation proceeds in three steps. First, a segmentation analysis was applied (via a cluster algorithm) to the subpopulation diagnosed with type 2 diabetes. A large number of variables associated with the social and behavioral determinants of health serve as the case attributes. The segmentation yields a minimum set of median profiles that characterize this subpopulation and uses the smallest number of attributes to do so. These profiles are then used to screen the population without diabetes for cases that most closely match them. This is roughly similar in intent to the marketer’s use of personal data from customers to identify future prospects. In this case, vulnerability is grounded in resemblance (in a unique variety of social and economic ways) to those with diabetes. The matching cases can be mapped based on geographical data to identify the neighborhoods with the highest relative concentration of cases and are the vulnerable (by resemblance) neighborhoods.

Next, the same set of variables used for segmentation analysis of the subpopulation with diabetes are subject to recursive partitioning over the entire population. The intent is to narrow down the set to the fewest, most accurate predictors of diabetes in the Houston area. As it turns out, the best population-level predictors are biological ones: factors such as blood pressure, body mass, and age. These factors can then be applied to the vulnerable neighborhoods, defined above, as a second layer of vulnerability related to predictive factors.

Finally, the more traditional notion of vulnerability, based on social and economic disadvantage, can be applied as a third layer and used to establish greater differentiation within the vulnerable neighborhoods. For a particular neighborhood designated as having the highest concentration of matchers vulnerable to diabetes, these cases can be further divided into those who share the same biological factors as the subpopulation with diabetes, and those who do not. A similar refinement can then be accomplished with measures of disadvantage. In this way, an ordered spectrum of vulnerability can be established within these neighborhoods. Those who have the predictive factors present, as well as disadvantage, fall further along the spectrum of vulnerability than do those who have only one or the other present. Those with neither, still have vulnerability by resemblance and represent an important (but distinctive) target group for preventive measures.

Importantly, this layered, nonparametric approach to vulnerability captures characteristics (and therefore individuals) that may be overlooked in a more limited analysis of a few factors. For example, a recent study in Cumberland County, New Jersey, examining how lifestyle behaviors and demographic characteristics influence individuals’ risk for developing diabetes, was limited to only a few demographic and socioeconomic characteristics—education levels below a four-year college degree, a household income of less than $50,000 per year, and age of 45 to 84 years—in part, due to the availability of these data at a population level [13].

The range of indicators in the cluster profiles supports the notion that type 2 diabetes spans races, ethnicities, and socioeconomic strata. This prevents stereotyping of those who might be most vulnerable; vulnerability as a composite concept works as a combination of factors working in concert. For example, individuals within cluster 1 were defined in part by having private insurance and living at or above 400% of the FPL. Those who match these profiles join the ranks of the vulnerable without necessarily having low socioeconomic status or facing barriers to accessing health care resources. The CDC reports that rates of diabetes increase as education levels decrease. And yet, in comparison with the decade from 1980 to 1990, the age-adjusted rates of diabetes since 1990 for those with a high school education have quadrupled, and for those with greater than a high school-level education have doubled [14]. It appears that higher socioeconomic status offers some protection against sharply increasing rates of the disease at a population level; however, the question remains whether identifying potentially vulnerable groups much earlier—prior to any clinical indications—will help reduce incidence rates over time. This study proposes a method for identifying these vulnerable groups, because no single social indicator will suffice. Further, attention to differentiating among the vulnerable, for example, by adopting our composite notion of layered vulnerability, should encourage more tailored, community-based interventions. The idea is to conserve and target resources through more effective identification of those highest on our vulnerability spectrum. 

## 5. Community Health Implications

The practice of using formative research conducted within communities to guide public health initiatives is successful in other fields as well [15]. For example, in many HIV (human immunodeficiency virus)-prevalent areas, programs have identified high-risk profiles within a larger population and implemented refined approaches to prevention in those communities [15]. The AIDS Community Demonstration Projects, for example, identified risk behaviors in a target population to guide the development of tailored educational materials [16]. Targeting the social environment as well as individual characteristics obtained via community-level data-gathering appears to be a uniquely effective method for community-based health action. 

The areas of vulnerability identified across Harris County will serve as a basis for further exploration through qualitative assessments and targeted prevention efforts. Although important large-scale public health interventions aimed at preventing diabetes exist, the most effective strategies for preventing new cases may be best implemented in a targeted fashion at the local level. The current trend in public health is heavily focused on community-based intervention to promote population-wide improvements in health [15]. However, not all community-centered programs are equally effective [17].

Studies examining best practices for enacting community-based health care initiatives have highlighted some important successes [17]. For example, within the diabetes field, several community-based initiatives have proved to be uniquely effective at improving diabetes outcomes. Partnerships within communities between patients, physicians, and health care systems reduce health disparities [18,19,20]. In addition, programs that employ culturally tailored, localized initiatives to combat diabetes improve health knowledge, behaviors, and a variety of diabetes outcomes. Culturally appropriate patient education, community outreach and partnerships, and case management with health care workers have been more effective at improving outcomes, compared with many general quality improvement interventions [21,22,23]. Redefining what it means to face the possibility of diabetes in the near or remote future is likely to change the way interventions are designed and targeted.

The CCD program is designed to gather data and implement tailored initiatives on a local level to prevent the rising rates of type 2 diabetes in urban settings. Our composite approach to vulnerability can assist studies in other urban centers to establish the best places to concentrate prevention resources. Neighborhoods found to be vulnerable by resemblance, and by prediction, across levels of disadvantage, can then be targeted with specialized interventions that aim to prevent type 2 diabetes far upstream. Subsequent findings on the social and cultural differences among the groups corresponding to the positions in Figure 5 will assist with this customization.

## 6. Conclusions

The approach taken here differs from clinical approaches for identifying groups “at risk” for diabetes or other chronic diseases in at least three ways. First, because this study focuses on primary prevention, our strategy is to identify those on the path to diabetes, without being warned of it by conventional screening measures. In this sense, they are “vulnerable” but not yet identified as at risk. Second, each particular path to diabetes is one complicated by socioeconomic and cultural factors that are seldom admitted to biomedical explanations of type 2 etiology. Accommodating these factors expands the focus on prevention beyond behavior modification and lifestyles to include the complicated relationship between opportunities for and barriers to change that are context specific. This approach emphasizes the social determinants of health as the key to reducing incidence rates beyond what current interventions have been able to produce. 

Third, although these results are considered as replicable and the techniques as generally applicable, the findings themselves are conditioned by the selected population. Most of the measures in this study rely only on the ordinal-scale properties in the data. The techniques used require no distributional assumptions. And all of the categorizations are made through relative comparisons only, that is, by selecting upper quartiles or ordering position or possessing two of three attributes, rather than relying on ratio-scaled amounts. Therefore, the vulnerable neighborhoods are vulnerable relative to all other neighborhoods in Harris County, and not in any absolute sense.

This latter feature is a strength when dealing with social factors but may be seen as a weakness in terms of conclusiveness or quantitative certainty. Inclusion of social determinants is still at an early stage that warrants a cautious approach to measurement, relying more on ordinal properties until measures can be scaled-up based on confidence in understanding their behavior. Another potential source of criticism is the logic behind profile matching based on clustering identifiers among people with type 2 diabetes. This profile method was used, in part, because there is no reason to assume that either the most appropriate set of indicators is already known, or that these indicators combine in a linear fashion to affect vulnerability. Finally, the composite notion of vulnerability assumes that this condition can have neighborhood features, which are perhaps emergent and difficult to measure, as well as personal-level features that combine to exacerbate the condition overall, but not necessarily in an additive way. Instead, the combination of features is conceptualized as working in layers, with each layer contributing to an assignment of residents along a spectrum of vulnerability. As with other notions, the spectrum is simply an array of relative positions in an ordered sequence. In this instance, we are able to identify subpopulations who are vulnerable in 1 layer but not in all 3, and include them in the customization of prevention efforts.

This level of differentiation is important in the context of population health because it allows for a more nuanced and comprehensive understanding of the prevention challenges. The experience of vulnerability across layers will differ, as will the social and cultural factors that reinforce these patterns. By understanding vulnerability as a composite notion, conventional prevention programming can be adapted to accommodate the variety of experiences at the local level, but also recognize that what is necessary for one group may not be required in another. 

## Figures and Tables

**Table 1:** Description of indicator categories within each cluster (unweighted data).

Indicator Category	Most Common Category (%)		
	Diabetes Cluster 1	Diabetes Cluster 2	Diabetes Cluster 3
**Race/Ethnicity**	***n* (%)**	***n* (%)**	***n* (%)**
White NH	65 (37.4)	71 (71.0)	11 (14.1)
Black NH	35 (20.1)	10 (10.0)	44 (56.4)
Hispanic	49 (28.2)	8 (8.0)	21 (26.9)
Other NH	25 (14.4)	11 (11.0)	2 (2.6)
**Age, years**			
20–44	22 (12.64)	0	3 (3.9)
45–54	61 (35.1)	0	20 (25.6)
55–64	88 (50.6)	4 (4.0)	26 (33.3)
65+	3 (1.7)	96 (96.0)	29 (37.2)
**Sex**			
Females	105 (60.3)	45 (45.0)	61 (78.2)
Males	69 (39.7)	55 (55.0)	17 (21.8)
**Total**	174 (49.4)	100 (28.4)	78 (22.2)
**Vulnerability Factor Indicator**	**Factor (%)**	**Factor (%)**	**Factor (%)**
Health insurance	Private (86.8)	Public (100)	Public (80.8)
Age, years	55–64 (50.6)	≥65 (96.0)	≥65 (37.2)
Public programs	None (98.3)	None (94.0)	≥1 (78.2)
Employment	Employed (78.2)	Unemployed (81.0)	Unemployed (98.7)
FPL	≥400% (48.9)	200%–399.9% (39.0)	<100% (64.1)
Difficulty buying food	Never (82.8)	Never (92.0)	Rarely/sometimes (50.0)
Days of poor health	0 (51.7)	0 (61.0)	≥8 (69.2)
Race/ethnicity	White (37.4)	White (71.0)	Black (56.4)
General health	Good (43.1)	Good (47.0)	Fair/poor (76.9)
Social support	Low (36.2)	Medium (38.0)	Low (55.1)
Physical activity level	Active/highly active (40.2)	Active/highly active (49.0)	Somewhat active (44.9)

FPL—Federal Poverty Level; NH—non-Hispanic.

## Appendix A

### Indicators Submitted to Cluster Analysis

Thirty-five economic, social, and physical indicators of health and well-being within the subpopulation living with type 2 diabetes: linguistic isolation, country of birth, age, sex, marital status, education level, race/ethnicity, employment, FPL, participation in public programs, difficulty buying food, difficulty paying rent/mortgage, general health status, number of days poor physical health, number of days unable to perform usual activities, serious psychological distress, HBP, fast food consumption, breakfast consumption, BMI, physical activity level, health insurance, health care provider, care type, problem paying medical bills, transport to medical visits, car use, household number of adults, household number of children, type of residence, time in the neighborhood, neighborhood stray animals, neighborhood fruits and vegetables availability, social support, neighborhood crime.
